# Smartphone-Based Self-Reports of Depressive Symptoms Using the Remote Monitoring Application in Psychiatry (ReMAP): Interformat Validation Study

**DOI:** 10.2196/24333

**Published:** 2021-01-12

**Authors:** Janik Goltermann, Daniel Emden, Elisabeth Johanna Leehr, Katharina Dohm, Ronny Redlich, Udo Dannlowski, Tim Hahn, Nils Opel

**Affiliations:** 1 Department of Psychiatry University of Münster Münster Germany; 2 Institute of Psychology University of Halle Halle Germany; 3 Interdisciplinary Centre for Clinical Research Münster University of Münster Münster Germany

**Keywords:** mobile monitoring, smartphone, digital biomarkers, digital phenotyping, course of illness, psychometric quality, mood disorders, depression, affective disorders, mobile phone

## Abstract

**Background:**

Smartphone-based symptom monitoring has gained increased attention in psychiatric research as a cost-efficient tool for prospective and ecologically valid assessments based on participants’ self-reports. However, a meaningful interpretation of smartphone-based assessments requires knowledge about their psychometric properties, especially their validity.

**Objective:**

The goal of this study is to systematically investigate the validity of smartphone-administered assessments of self-reported affective symptoms using the Remote Monitoring Application in Psychiatry (ReMAP).

**Methods:**

The ReMAP app was distributed to 173 adult participants of ongoing, longitudinal psychiatric phenotyping studies, including healthy control participants, as well as patients with affective disorders and anxiety disorders; the mean age of the sample was 30.14 years (SD 11.92). The Beck Depression Inventory (BDI) and single-item mood and sleep information were assessed via the ReMAP app and validated with non–smartphone-based BDI scores and clinician-rated depression severity using the Hamilton Depression Rating Scale (HDRS).

**Results:**

We found overall high comparability between smartphone-based and non–smartphone-based BDI scores (intraclass correlation coefficient=0.921; *P*<.001). Smartphone-based BDI scores further correlated with non–smartphone-based HDRS ratings of depression severity in a subsample (*r*=0.783; *P*<.001; n=51). Higher agreement between smartphone-based and non–smartphone-based assessments was found among affective disorder patients as compared to healthy controls and anxiety disorder patients. Highly comparable agreement between delivery formats was found across age and gender groups. Similarly, smartphone-based single-item self-ratings of mood correlated with BDI sum scores (*r*=–0.538; *P*<.001; n=168), while smartphone-based single-item sleep duration correlated with the sleep item of the BDI (*r*=–0.310; *P*<.001; n=166).

**Conclusions:**

These findings demonstrate that smartphone-based monitoring of depressive symptoms via the ReMAP app provides valid assessments of depressive symptomatology and, therefore, represents a useful tool for prospective digital phenotyping in affective disorder patients in clinical and research applications.

## Introduction

The phasic development of symptoms over time in the form of disease episodes is one of the key characteristics of affective disorders. These disease trajectories can be used as an informative predictor as well as an outcome measure in psychiatric research and personalized medicine. However, the assessment of the development of symptoms over time is challenging. The value of cross-sectional assessments is limited as they can only capture an excerpt of the symptom history and it is unclear whether this excerpt reflects, for example, the peak of an affective episode or a fully or partially remitted state and whether episodes are recurrent. Collecting this information retrospectively from the patients is one approach to gaining insights into their former symptom history, which is likely to be biased by their current depressive state [1]. Thus, multiple prospective assessments of symptoms are needed for a valid interpolation of the underlying disease trajectory. Although such prospective instruments based on a paper-and-pencil format exist [2], their use is limited due to low cost-efficiency as well as low patient compliance [3]. In recent years, the utilization of smartphone apps for psychological and psychiatric assessment has increased considerably due to the cost-efficiency and practicability of these apps [4-6]. 

Several proof-of-concept studies have pointed to the utility of smartphone-based data in affective disorder research [7]. Smartphone-based measures can be categorized into passive sensor data (eg, geolocation, distance, steps, acceleration, and app activities) and active self-report. The latter, which entails daily diaries, reiterated questionnaires, and ecological momentary assessments, utilizes multiple assessments per day, thereby acquiring different micro- or macrolevels of affective symptomatology [8]. The focus of this paper is the assessment and validation of active self-report data.

The potential of continuous monitoring of psychomotor activity based on acceleration and location for a differentiation of unipolar and bipolar patients has been demonstrated [9,10]. Recent studies have also indicated that smartphone-based movement parameters allow for a prediction of intraindividual, daily mood state changes [4,11-14]. However, such prospective investigations require in-depth knowledge of the psychometric properties of the acquired data especially when it comes to the validity of smartphone-based measurements. This point appears particularly important in study designs that entirely rely on smartphone-based data.

Consequently, the comparability between smartphone-based and non–smartphone-based versions (ie, conventional paper-and-pencil or stationary computer-based versions) of psychometric instruments has also received increasing attention [15]. Besides the obvious difference in the format in which content is presented, differences in the assessment setting (ie, laboratory or clinical setting vs variable situations in real life) as well as technical reservations could lead to different assessment results. Particularly when using smartphones, potential distractions may become more likely, with the environments of reporting participants being less controllable. Initial evidence suggests that scores derived from digital and paper-and-pencil psychometric instruments seem to be generally comparable, however, with considerable variance in the agreement [16-18]. Yet, a considerable number of previous studies investigating the reliability and validity of digital phenotyping methods have focused on computer-based assessments that might differ from mobile assessments via the participants’ smartphones as outlined above. For the Beck Depression Inventory (BDI), interformat reliability between non–smartphone-based paper-and-pencil versions and computer-based versions has been demonstrated across several studies [16], while large-scale validation reports of agreement between smartphone-based and non–smartphone-based versions are currently lacking.

Data from pilot studies indicate agreement between smartphone-delivered, daily self-rated mood and clinician-rated mood via Hamilton Depression Rating Scale (HDRS) scores among bipolar patients [19]; in addition, Juengst et al demonstrated high comparability between mood-related symptoms among traumatic brain injury patients assessed either via smartphone self-reports or via telephone interview [20]. In a systematic review of the literature including data from three studies and a total of 89 bipolar outpatients, significant medium-sized correlations between daily, smartphone-based self-report assessments of depressive symptoms and established clinical rating scales were reported [21]. Regarding smartphone-based monitoring in major depression, Torous et al reported high agreement between daily, smartphone-based self-reports and paper-and-pencil assessments using the Patient Health Questionnaire-9 (PHQ-9) among 13 adult patients with major depressive disorder (MDD) [22]; similarly, Cao et al reported agreement between daily, smartphone-based self-reported mood and the PHQ-9 among 13 adolescent participants [23]. One systematic review that investigated the psychometric properties of mobile mood monitoring among young people concluded that there is enormous heterogeneity in the validity of smartphone-based delivery formats and more high-quality studies are needed [15].

In sum, while the aforementioned findings of overall agreement between smartphone-based self-reported depressive symptoms and established clinical scales is encouraging, it appears important to denote that limited sample sizes in previous reports as well as systematic differences, including sample properties, technical properties, and assessment type, currently limit our understanding of the reliability and validity of smartphone-based assessments of depressive symptoms. It thus remains unclear to what degree validation reports of smartphone-based self-reports are generalizable across assessment instruments, cohorts, and applications; hence, app- or study-specific validation of measurements remains the gold standard.

Therefore, the aim of this study is to assess the validity of smartphone-based assessments of depressive symptoms using the Remote Monitoring Application in Psychiatry (ReMAP) app. To this end, we use smartphone-based depression self-reports using single-item and BDI questionnaire data and investigate their comparability with non–smartphone-based versions of the BDI, a well-established and standardized self-report instrument used among psychiatric patients and healthy control participants. We test the hypotheses that both delivery formats—smartphone-based and non–smartphone-based assessments—yield comparable results and, therefore, that smartphone-based monitoring of depressive symptoms via the ReMAP app provides valid assessments of depressive symptomatology. Furthermore, we aim to investigate potential differences in the agreement between smartphone-based and non–smartphone-based assessments of depressive symptoms across diagnostic groups as well as across age and gender.

## Methods

### Participants

The ReMAP study was designed as a prospective, naturalistic observational study. An overall sample of 173 participants was included in the analyses; participants had a mean age of 30.14 years (SD 11.92). The single inclusion criterion for this study was availability of a smartphone-based BDI that was completed within 4 weeks of a non–smartphone-based BDI. The sample included adults that were either healthy controls (n=101) or belonged to one of the following diagnostic groups: MDD (n=43), bipolar disorder (n=5), MDD with comorbid social anxiety disorder (SAD) (n=9), SAD only (n=2), or specific phobia (SP), spider subtype (n=13). Participants were recruited for ReMAP participation in the context of ongoing longitudinal cohort studies over which assessments were parallelized; details on subsamples from all cohorts are provided in the Multimedia Appendix 1.

Participants were informed about the possibility of voluntary additional participation in the ReMAP study in a face-to-face meeting at the time they presented at the Department of Psychiatry, University of Münster, Germany, in the context of ongoing, longitudinal cohort assessments. Interested subjects were extensively briefed about aims; methods, especially type and amount of collected data; details on data security (ie, details on data transfer and storage); and financial compensation. The study was approved by the local Institutional Review Board, and written informed consent was obtained before participation.

### Non–Smartphone-Based Measures and Procedures

All measures that were not assessed via smartphone (ie, conventionally administered in interviews or via paper-and-pencil or tablet questionnaires) will be referred to as *non–smartphone-based assessments* and are described below. Presence or absence of a psychiatric diagnosis was assessed in all participants via the Structured Clinical Interview for DSM-IV (Diagnostic and Statistical Manual of Mental Disorders, Fourth Edition) Axis I Disorders (SCID-I) [24,25] prior to participation in the ReMAP study. All healthy control participants were free from any history of a psychiatric disorder. As part of the original study assessments, participants from all cohorts provided self-reports of depressive symptoms via the BDI-I [26] or the BDI-II [27]. Both versions of the BDI are standardized and valid instruments for the assessments of depressive symptoms and represent well-established assessment tools in research and clinical routines for assessing the presence and extent of depressive symptoms. Additional assessments of clinician-rated depression severity via the HDRS [28] were available for a subset of 51 participants.

### The ReMAP Smartphone App

Development of ReMAP began in mid-2018 at the Institute for Translational Psychiatry in Münster. It is a native app for iOS and Android, based on Apple ResearchKit, Apple Health, and Google Fit. After an anonymous log-in with a provided subject ID, the app works in background mode and monitors the number of steps taken by the user, the distance walked, the accelerometer, and GPS position data. The data are encrypted on the smartphone and sent regularly via REST-API (REpresentational State Transfer application programming interface) to a back end specifically developed for ReMAP, which is provided on university servers. In addition, the app regularly enables the user to fill out various questionnaires regarding sleep and mood as well as to create short voice recordings. Measures used in this study’s analyses are described below.

### Smartphone-Based Measures and Procedures

After written informed consent was obtained, each participant was provided an individual subject ID (ie, subject code). The participant was then asked to download the developed ReMAP smartphone app and to start the app. At this time, subjects were asked to confirm participation in the study again and to enter their individual subject IDs.

In addition to the continuous assessment of passive data, all participants were asked to provide self-reported ratings of depressive symptoms. To this end, participants filled out a digital version of the BDI-I that was integrated into ReMAP every 2 weeks. Moreover, participants rated their mood and sleep duration by answering single items every 3 days. For the single mood question (ie, “How is your mood today?”), participants provided their responses via touch screen on a scale from 1 (very bad) to 10 (very good). For the single sleep question (ie, “How many hours did you sleep last night?”), participants provided their response on a scale from 0 to 13 hours. For all self-reported data, the app sent out weekly push notifications on a random basis during the daytime with a variance of 2 days or every 2 weeks in case of the BDI. The time of the day when notifications were sent was systematically varied in order to avoid bias from systematically assessing symptom self-reports (eg, only during the morning). Participants were instructed that answering all questions was optional and they were free to choose their time of answering whenever items were made available.

Again, for this study, smartphone-based and non–smartphone-based data were only included if the time interval between completion of the ratings between both delivery formats was less than 4 weeks, in order to minimize potential bias due to temporal change in depressive symptoms. Further, for each participant, the respective BDI, mood, and sleep assessments from the time point with the shortest interval between smartphone-based and non–smartphone-based assessments were included for this study.

### Statistical Analyses

Agreement between non–smartphone-based and smartphone-based BDI scores was assessed by absolute agreement using a two-way, mixed-effects intraclass correlation coefficient (ICC) [29]. To this end, the non–smartphone-based measures were compared with the temporally closest smartphone-based BDI scores available, resulting in the shortest interval possible.

This analysis was further repeated for the over-1-week-interval and the under-1-week-interval groups separately in order to assess the influence of the test-retest interval on the agreement between measurements. In addition, the analysis was repeated separately among healthy controls, affective disorder (ie, MDD, SAD + MDD, and bipolar disorder) patients, and anxiety disorder (ie, SP, spider subtype; and SAD) patients, as well as for the two non–smartphone-based BDI versions (ie, BDI-I and BDI-II). The internal consistency of the smartphone-based BDI was assessed via Cronbach α and compared with the internal consistency of the non–smartphone-based BDIs.

The smartphone-based single mood item was correlated with the non–smartphone-based and smartphone-based BDI scores. Although it covers different levels of symptomatology (ie, the BDI assesses complex symptoms over time, while the single mood item assesses only the current subjective mood [8]), the BDI questionnaire was used for validation based on the assumption that both measures are sensitive for current mood.

For validation of the smartphone-based single sleep item, it was correlated with the smartphone-based and non–smartphone-based BDI item assessing sleeping disturbance. Analogous to the BDI analysis, one mood and one sleep assessment were used for analysis based on the shortest interval to the non–smartphone-based measures. For further validation, the ReMAP BDI and the ReMAP single mood item were both correlated with clinician-rated depression severity using the HDRS.

All analyses were conducted using SPSS, version 26 (IBM Corp). A multiple test correction was undertaken across all significance tests (n=34) in order to avoid α error accumulation using a false-discovery-rate (FDR) correction following the Benjamini-Hochberg procedure [30]. Assuming an FDR q value of .05, this approach yielded a corrected significance threshold of *P*<.04.

## Results

### Descriptive Statistics

Mean BDI scores and their range across all participants were similar for ReMAP (mean 5.35, SD 8.63; range 0-44) and non–smartphone-based BDI (mean 6.46, SD 9.06; range 0-47). Absolute differences between both measurements were, on average, 3.02 points (SD 3.76) with a considerable range covering 0 to 26 points. The mean test-retest interval was 5.84 days (SD 7.29), ranging from 0.20 to 28.70 days. Detailed descriptive statistics across subgroups of the sample are provided in Table S1 in Multimedia Appendix 1. Among the included participants who completed a smartphone-based BDI within 4 weeks of completing non–smartphone-based measures, the percentages of participants who also provided single items for mood and sleep within a maximum interval of 4 weeks were 97.11% and 95.95%, respectively.

### Validity of Affective Symptom Assessment via ReMAP

**Figure 1:**
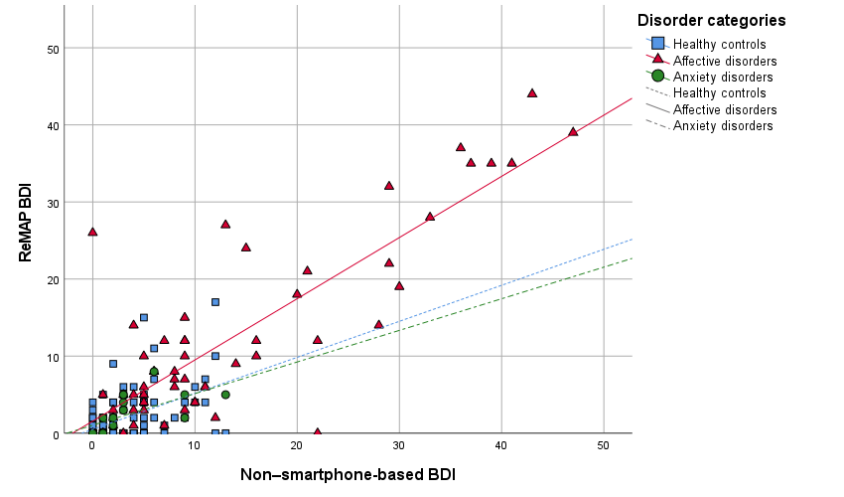
Beck Depression Inventory (BDI) scores via the Remote Monitoring Application in Psychiatry (ReMAP) smartphone app over non–smartphone-based BDI scores across diagnostic groups.

The overall agreement between ReMAP and the non–smartphone-based BDI was very high (ICC 0.921, 95% CI 0.890-0.942). Separate investigations of the BDI agreement in several subgroups yielded highly comparable ICCs across both BDI versions (ie, BDI-I and BDI-II), across different test-retest intervals, across different age groups, and across males and females—the ICC was over 0.888 for all subgroups. Separate investigations across different diagnostic statuses yielded the highest BDI agreement between delivery formats in the subgroup with affective disorders (ICC 0.912), while healthy controls and participants with anxiety disorders (ie, SP, spider subtype; and SAD) showed moderate agreement between BDIs (ICC 0.639 and ICC 0.736, respectively). Similarly, higher agreement was found among acutely depressed as compared to remitted MDD patients (see Multimedia Appendix 1). ICC statistics for the full sample and all subgroups are presented in Table 1. Scatterplots of ReMAP BDI scores over non–smartphone-based BDI scores are provided in Figure 1.

The internal consistency of the ReMAP BDI (Cronbach α=.944, n=174) was virtually identical to both non–smartphone-based BDI versions (BDI-I: α=.945, n=54; BDI-II: α=.944, n=108). For further validation, the ReMAP BDI was correlated with clinician-rated depression severity using the HDRS in a subset of the sample (n=51). The analysis yielded a strong significant correlation (*r*=0.783; *P*<.001) that was comparable to the association between the HDRS score and the score of the non–smartphone-based BDI (*r*=0.682; *P*<.001).

**Table 1:** Intraclass correlation agreement of the Remote Monitoring Application in Psychiatry (ReMAP) Beck Depression Inventory-I (BDI-I) with the full sample and stratified subsamples.

Sample	Number of participants (N=173), n (%)	Intraclass correlation coefficient	95% CI	*P* value^a^
Full sample	173 (100)	0.921	0.890-0.942	<.001
BDI-I_non–smartphone based_	64 (37.0)	0.921	0.870-0.952	<.001
BDI-II_non–smartphone based_	109 (63.0)	0.919	0.863-0.850	<.001
≤1-week interval^b^	126 (72.8)	0.934	0.890-0.958	<.001
>1-week interval^c^	47 (27.2)	0.888	0.799-0.938	<.001
Healthy controls	101 (58.4)	0.639	0.454-0.760	<.001
Affective disorders	57 (32.9)	0.912	0.851-0.948	<.001
Anxiety disorders	15 (8.7)	0.736	0.252-0.910	.008
Age ≤35 years	131 (75.7)	0.899	0.851-0.931	<.001
Age >35 years	42 (24.3)	0.962	0.930-0.980	<.001
Male	41 (23.7)	0.969	0.919-0.986	<.001
Female	132 (76.3)	0.904	0.864-0.933	<.001

^a^All *P* values below a false discovery rate–corrected significance threshold of *P*<.04 are considered statistically significant.

^b^Participants completed the smartphone-based BDI within 1 week of completing non–smartphone-based measures.

^c^Participants completed the smartphone-based BDI and non–smartphone-based measures more than 1 week apart.

After including all data points with a test-retest interval of up to 4 weeks, the single item for mood assessed via ReMAP correlated moderately with the sum scores of the ReMAP BDI (*r*=–0.538; *P*<.001; n=168) and with both non–smartphone-based BDI versions (BDI-I: *r*=–0.485, *P*<.001, n=61; BDI-II: *r*=–0.504, *P*<.001, n=107). Further, a significant negative correlation between the ReMAP single mood item and the HDRS score was observed (*r*=–0.369; *P*=.008; n=51). Correlations of the single mood item across subsamples are provided in Table S2 in Multimedia Appendix 1.

The single item for sleep assessed via ReMAP was correlated with the BDI item assessing sleeping disturbance. After including all data points with test-retest intervals of up to 4 weeks, this analysis yielded significant negative associations with the sleep item from the ReMAP BDI (*r*=–0.310; *P*<.001; n=166) and with the sleep item of both non–smartphone-based BDI versions (BDI-I: *r*=–0.279, *P*=.03, n=63; BDI-II: *r*=–0.202, *P*=.04, n=102). Separate correlation analyses of the single mood and sleep ReMAP items across disorder subgroups are presented in Table S3 in Multimedia Appendix 1. The general pattern of results yielded the strongest associations in the affective disorder group.

## Discussion

With this study, we demonstrate that smartphone-based monitoring of depressive symptoms via the ReMAP app provides valid assessments of depressive symptomatology. The overall high agreement between the non–smartphone-based and smartphone-based versions of the BDI confirm that digital assessments via the ReMAP app using the participants’ smartphones have the potential to offer valid estimates of the trajectory of participants’ moods. This notion is additionally supported by the observed correlation of smartphone-administered single-item ratings regarding mood and sleep with corresponding non–smartphone-based assessments. Importantly, the validity of smartphone-based assessments could furthermore be demonstrated by using clinical rating scales as a criterion with a strong correlation of smartphone-based BDI and non–smartphone-based HDRS scores.

The observation of high agreement between self-reported smartphone-based assessments of depressive symptoms and classic non–smartphone-based assessments in this study is supported by previous findings from pilot studies among MDD patients [22] and from a systematic review among bipolar patients [21]. Furthermore, the comparability of the non–smartphone-based and smartphone-based versions of the BDI in our study matches similar results of agreement between paper-and-pencil and computer versions of the BDI [16].

Our findings of overall high validity of smartphone-based and conventional non–smartphone-based assessments of depressive symptoms in a relatively large and heterogeneous sample critically underscores the potential of mobile assessment tools in psychiatric research. Considering that smartphone-based assessments offer valid data on patients’ mood states, an expansion of mobile data acquisition in the clinical and research context appears desirable. The cost-efficiency of smartphone-based data might thus allow the acquisition of valid data on patients’ long-term disease trajectories at an unprecedented scale. Together with previous studies investigating the comparability of the BDI versions (ie, BDI-I and BDI-II) [27,31] as well as delivery formats [16], our findings add to an increasing evidence base of high comparability of smartphone-based and conventional non–smartphone-based assessments of depressive symptoms.

We furthermore demonstrate that agreement between smartphone-based and non–smartphone-based assessments of depressive symptoms does not depend on the age or gender of participants, which supports the generalizability of smartphone-based assessments of depressive symptoms. This notion appears especially noteworthy considering the relatively large sample size, in comparison with previous reports, as well as the age range of participants included in this study (ie, 18-68 years of age). The inclusion of older participant groups seems relevant, as previous studies have emphasized that smartphone apps for mental health monitoring should meet the needs (eg, easy handling) of older and potentially less technically proficient individuals in order to assure adherence among these group members [32].

An important observation of this study was that higher agreement between smartphone-based and non–smartphone-based assessments of depressive symptoms was found among affective disorder patients compared to anxiety disorder patients or healthy controls. Notably, while the agreement in the affective disorder sample can be estimated as excellent, intraclass correlations indicate a lower, but still moderate to good, agreement in the healthy control and anxiety disorder samples [33]. This might partly be traced back to the much higher variance of depression severity in the affective disorder group. Lower variance in depression scores in nonaffective clinical samples has previously been suggested to account for findings of low reliability among substance addiction patients [34]. Further, small sample sizes of some participant subgroups limit the weight of this finding, particularly for the anxiety disorder subgroup (n=15). These findings may call for a cautious interpretation of findings based on self-reported symptom data in healthy or nonaffective disorder populations. However, they also seem to contradict previous findings. The authors of a meta-analysis investigating BDI reliability concluded that nonclinical samples show a very good test-retest reliability, while only very limited data are available for test-retest reliability in clinical samples [31]. Sporadic reports of lower retest reliabilities as found by one study [35] were explained by the authors as natural changes in depression severity over time [31]. The difference in reliabilities across samples may, in part, stem from differences in statistical analyses, as traditional Pearson correlations that were used by the cited studies can produce substantially different results than ICC agreement estimates, which are now often recommended for retest analysis [36].

Besides validation of a smartphone version of the BDI, this study found moderate to high agreement between mood ratings via smartphone-based single-item assessments and established clinical scores using the BDI, regardless of the delivery format of the BDI. This finding is of particular importance considering that completion of an entire questionnaire is time-consuming and, hence, the usage of single items might provide a valid possibility of assessing mood on a frequent basis. Importantly, these findings are tentative and limited by the fact that the single mood item and the BDI questionnaires may systematically assess differing concepts in regard to the symptom level as suggested by previous scholars [8]. However, although this distinction may account for agreement between both measures, the high agreement also points to substantial overlap between the macrolevel BDI questionnaire and the more microlevel single mood item.

In sum, the associations between questionnaire data (ie, the BDI) and single-item mood self-reports pose the following question: Which measure may be better suited for specific research contexts and could one of the two be omitted completely? One may argue that single mood items seem to provide a sufficient proxy for the assessment of mood fluctuations that is more time-efficient and could, therefore, be assessed more frequently as compared to a more exhaustive BDI questionnaire. On the other hand, it may be more beneficial to have a more elaborate symptom profile as obtained, for example, via the BDI in exchange for assessment frequency. It remains to be investigated what temporal and content-related resolution is most beneficial for the investigation of the development of depressive symptoms and for specific feature engineering using machine learning algorithms. Likely, the most beneficial trade-off between the two highly depends on the specific research question.

Compared with the single mood item, the single sleep item showed a lower correlation with corresponding non–smartphone-based assessments in the form of sleep disturbance items within the BDI questionnaire. One possible explanation for this low association is that both items measure slightly different aspects of sleep: while the BDI sleep item assesses increased and decreased sleep duration and, depending on the BDI version, also a combination with subjective sleep quality, the single sleep item assesses purely the duration of sleep during the last night. Further, sleep quality or disturbance may be a more heterogeneous construct and, thus, more difficult to assess via a single item. Another possible explanation for this finding could be that variability in the sleep quality, as well as sleep duration, may be less temporally stable as compared to mood changes. Thus, the test interval of up to 4 weeks may be too long in order to validate the smartphone-based assessment of sleep duration. Considering that smartphone-based and non–smartphone-based assessment methods lie several days or weeks apart, the association between them seems to be reasonably high.

Strengths of this study include the relatively large sample of participants and the availability of smartphone-based data along with conventional psychometric and clinical data. Furthermore, this study included participants with differing psychiatric diagnoses and a high variability in age, thus allowing the assessment of generalizability across such participant groups. Further, a wide variety of assessment forms were used for validation, considering multiple sources of information. The application of non–smartphone-based BDI versions (ie, self-report), as well as clinical ratings (ie, HDRS), underlines the validity of the smartphone-based assessments via the ReMAP app. Limitations include the lack of prospective clinical follow-up data. Future large-scale studies are warranted to assess the prognostic validity of smartphone-based self-reports in affective disorder patients.

Smartphone-based monitoring of depressive symptoms remains a timely matter of critical relevance for translational psychiatry. These results demonstrate overall high validity of smartphone-based assessments of depressive symptoms and should, thus, encourage researchers to apply mobile apps toward continuous prospective assessments of depressive symptoms.

## Appendix

Multimedia Appendix 1 Descriptive statistics of subsamples, correlations of the Remote Monitoring Application in Psychiatry (ReMAP) single mood item with Beck Depression Inventory (BDI) scores across subsamples, and correlations of the ReMAP single sleep item with the BDI sleep item across subsamples.

## Abbreviations

BDI: Beck Depression Inventory
CRC-TRR: Collaborative Research Center Transregio
DFG: German Research Foundation
DSM-IV: Diagnostic and Statistical Manual of Mental Disorders, Fourth Edition
FDR: false discovery rate
HDRS: Hamilton Depression Rating Scale
ICC: intraclass correlation coefficient
IMF: Innovative Medizinische Forschung
IZKF: Interdisciplinary Center for Clinical Research
MDD: major depressive disorder
PHQ-9: Patient Health Questionnaire-9
ReMAP: Remote Monitoring Application in Psychiatry
REST-API: REpresentational State Transfer application programming interface
SAD: social anxiety disorder
SCID-I: Structured Clinical Interview for DSM-IV Axis I Disorders
SP: specific phobia
