# Supplementary material for: Smartphone-Based Self-Reports of Depressive Symptoms Using the Remote Monitoring Application in Psychiatry (ReMAP): Interformat Validation Study
Source: JMIR Ment Health. 2021 Jan 12;8(1):e24333. doi: 10.2196/24333 (PMC7837996; doi:10.2196/24333)
Supplement: Multimedia Appendix 1 [file mental_v8i1e24333_app1.docx]

**Online Supplements**

*Description of sample cohorts*

*Survey instructions that patients have received for the mobile version*

*Table S1. Descriptive statistics over subsamples.*

*Table S2. Correlations of ReMAP single mood item with BDI scores over subsamples.*

*Table S3. Correlations of ReMAP single sleep item with BDI sleep item over subsamples.*

*Description of sample cohorts*

The sample for the current analyses were drawn from several ongoing longitudinal cohorts. Included samples stem from the Marburg/MünsterAffectiveDisorderCohortStudy (MACS, n=47) (Vogelbacher et al., 2018), the MünsterNeuroimageCohort (MNC, n=17) (Dannlowski et al., 2016; Opel et al., 2019), two subsamples of the SFB-TRR58 cohort (n=81; Z02 Münster cohort and SpiderVR Münster cohort (Schwarzmeier et al., 2020)), and the TIP (n=28) cohort. All cohorts comprise healthy control (HC) participants, as well as different patient groups. Both, the MACS and the MNC cohorts include major depressive disorder (MDD) and bipolar disorder (BD) patients under current or former inpatient treatment. The SFB-TRR58 sample includes patients with a spider phobia (SP). The TIP sample includes patients with a social anxiety disorder (SAD), MDD, or with comorbid SAD and MDD.

*Supplementary Results*

In order to investigate if low BDI scores in a substantial proportion of the sample of MDD participants drives the high agreement between smartphone-based and non–smartphone-based BDI scores, acute and remitted MDD patients were analyzed separately. We defined all MDD participants with a BDI score of 10 or greater as acutely depressed, while BDI scores below that were defined as remitted. This analysis yielded that *the participants with acute depressive symptoms show higher interformat agreement* (ICC=.865, p<.001, n=26), as compared to the affective disorder patients with no or subclinical depression symptoms (ICC=.326, p=.103, n=31). Although this finding should be interpreted with caution due to the low sample sizes, it indicates that high interformat agreement in affective disorders is not driven by participants with low levels of depressive symptoms.

*Survey instructions for the smartphone-based self-reports*

Beck Depression Inventory

*German:* „Dieser Fragebogen enthält 21 Gruppen von Aussagen. Bitte lesen Sie jede Gruppe sorgfältig durch. Suchen Sie dann die eine Aussage in jeder Gruppe heraus, die am besten beschreibt, wie Sie sich in dieser Woche einschließlich heute gefühlt haben und kreuzen Sie die dazugehörige Ziffer (0, 1, 2 oder 3) an. Lesen Sie auf jeden Fall alle Aussagen in jeder Gruppe, bevor Sie Ihre Wahl treffen.“

*English (translated):* This questionnaire contains 21 groups of statements. Please read each group carefully. Then pick out the one statement in each group that best describes how you felt this week and today and mark the corresponding number (0, 1, 2 or 3). Be sure to read all the statements in each group before making your choice.

Mood Item

*German:* „Wie ist Ihre Stimmung heute?“ 1 (sehr schlecht) – 10 (sehr gut)

*English:* „How is your mood today?” 1 (very bad) - 10 (very good)

Sleep Item

*German:* „Wie viele Stunden haben Sie in der letzten Nacht geschlafen?“ 0 – 13

*English:* "How many hours did you sleep last night?" 0 - 13

For the mood and sleep item no additional instructions were provided.

**Table S1.** Descriptive statistics over subsamples.

|  | Age | | Sex | BDI_ReMAP_ | | BDI_non–smartphone-based_ | | BDI difference (absolute) | | Intervall (days) | | Mood_ReMAP_ | | Sleep_ReMAP_ | |
| --- | --- | --- | --- | --- | --- | --- | --- | --- | --- | --- | --- | --- | --- | --- | --- |
|  | M (SD) | range | m/f | M (SD) | range | M (SD) | range | M (SD) | range | M (SD) | range | M (SD) | range | M (SD) | range |
| Full sample (n=173) | 30.14  (11.92) | 18-68 | 41/132 | 5.35  (8.63) | 0-44 | 6.46  (9.06) | 0-47 | 3.02  (3.76) | 0-26 | 5.84  (7.29) | 0.20-28.70 | 7.25  (1.67) | 2-10 | 7.30  (1.51) | 2-11 |
| *BDI version subgroups* |  |  |  |  |  |  |  |  |  |  |  |  |  |  |  |
| BDI-I_non–smartphone-based_ (n=64) | 37.17  (14.28) | 20-68 | 14/50 | 7.20  (10.54) | 0-44 | 7.48  (10.18) | 0-43 | 3.25  (4.61) | 0-26 | 8.07  (8.07) | 0.20-28.70 | 6.91  (1.87) | 2-10 | 7.22  (1.68) | 2-10 |
| BDI-II_non–smartphone-based_ (n=109) | 26.02  (7.81) | 18-58 | 27/82 | 4.26  (7.12) | 0-39 | 5.85  (8.32) | 0-47 | 2.88  (3.18) | 0-14 | 4.52  (6.48) | 0.40-28.60 | 7.46  (1.51) | 3-10 | 7.36  (1.40) | 3-11 |
| *Retest interval subgroups* |  |  |  |  |  |  |  |  |  |  |  |  |  |  |  |
| <1 week interval (n=126) | 28.79  (10.72) | 18-58 | 30/96 | 4.59  (8.16) | 0-44 | 6.18  (9.08) | 0-47 | 2.77  (3.35) | 0-22 | 1.82  (1.91) | 0.20-7.80 | 7.33  (1.64) | 2-10 | 7.26  (1.50) | 2-10 |
| >1 week interval (n=47) | 33.77  (14.17) | 18-68 | 11/36 | 7.38  (9.57) | 0-37 | 7.19  (9.08) | 0-41 | 3.68  (4.66) | 0-26 | 16.59  (5.15) | 8.40-28.70 | 7.04  (1.75) | 3-10 | 7.43  (1.53) | 4-11 |
| *Disorder subgroups* |  |  |  |  |  |  |  |  |  |  |  |  |  |  |  |
| healthy control (n=101) | 28.92  (11.52) | 18-68 | 26/75 | 1.89  (3.12) | 0-17 | 2.97  (3.30) | 0-13 | 2.25  (2.55) | 0-13 | 5.14  (6.83) | 0.20-28.70 | 7.67  (1.31) | 4-10 | 7.36  (1.27) | 3-10 |
| affective disorders (n=57) | 33.61  (12.73) | 18-66 | 14/43 | 12.23  (11.74) | 0-44 | 13.42  (12.45) | 0-47 | 4.70  (5.08) | 0-26 | 7.72  (7.91) | 0.30-27.60 | 6.33  (1.90) | 2-10 | 7.26  (1.88) | 2-11 |
| anxiety disorders (n=15) | 25.20  (8.06) | 18-43 | 1/14 | 2.47  (2.47) | 0-8 | 3.47  (4.00) | 0-13 | 1.80  (2.57) | 0-8 | 3.33  (6.68) | 0.40-22.30 | 8.07  (1.44) | 5-10 | 7.07  (1.49) | 4-10 |

**Table S2.** Correlations of ReMAP single mood item with BDI scores over subsamples.

|  | *r* | *P* | n |
| --- | --- | --- | --- |
| *All disorder groups* |  |  |  |
| BDI-I_ReMAP_ | -0.538 | <.001 | 168 |
| BDI-I_non–smartphone-based_ | -0.485 | <.001 | 61 |
| BDI-II_non–smartphone-based_ | -0.504 | <.001 | 107 |
| *Healthy controls* |  |  |  |
| BDI-I_ReMAP_ | -0.317 | .001 | 98 |
| BDI-I_non–smartphone-based_ | -0.170 | .405 | 26 |
| BDI-II_non–smartphone-based_ | -0.337 | .004 | 72 |
| *Affective disorders* |  |  |  |
| BDI-I_ReMAP_ | -0.494 | <.001 | 58 |
| BDI-I_non–smartphone-based_ | -0.462 | .005 | 35 |
| BDI-II_non–smartphone-based_ | -0.319 | .138 | 23 |
| *Anxiety disorders* |  |  |  |
| BDI-I_ReMAP_ | -0.092 | .776 | 12 |
| BDI-I_non–smartphone-based_ | - | - | - |
| BDI-II_non–smartphone-based_ | -0.657 | .020 | 12 |

***Note.*** *All p-values below a FDR-corrected significance threshold of p<.035 are considered statistically significant.*

**Table S3.** Correlations of ReMAP single sleep item with BDI sleep item over subsamples.

|  | *r* | *P* | n |
| --- | --- | --- | --- |
| *All disorder groups* |  |  |  |
| BDI-I_ReMAP_ sleep item | -0.310 | <.001 | 166 |
| BDI-I_non–smartphone-based_ sleep item | -0.279 | .027 | 63 |
| BDI-II_non–smartphone-based_ sleep item | -0.202 | .042 | 102 |
| *Healthy controls* |  |  |  |
| BDI-I_ReMAP_ sleep item | -0.387 | <.001 | 97 |
| BDI-I_non–smartphone-based_ sleep item | -0.179 | .371 | 27 |
| BDI-II_non–smartphone-based_ sleep item | -0.151 | .211 | 70 |
| *Affective disorders* |  |  |  |
| BDI-I_ReMAP_ sleep item | -0.395 | .002 | 57 |
| BDI-I_non–smartphone-based_ sleep item | -0.299 | .077 | 36 |
| BDI-II_non–smartphone-based_ sleep item | -0.379 | .090 | 21 |
| *Anxiety disorders* |  |  |  |
| BDI-I_ReMAP_ sleep item | 0.275 | .387 | 12 |
| BDI-I_non–smartphone-based_ sleep item | - | - | - |
| BDI-II_non–smartphone-based_ sleep item | -0.450 | .165 | 11 |

***Note.*** *All p-values below a FDR-corrected significance threshold of p<.035 are considered statistically significant.*

**References:**

Dannlowski, U., Kugel, H., Grotegerd, D., Redlich, R., Opel, N., Dohm, K., … Baune, B. T. (2016). Disadvantage of Social Sensitivity: Interaction of Oxytocin Receptor Genotype and Child Maltreatment on Brain Structure. *Biological Psychiatry*, *80*(5), 398–405. https://doi.org/10.1016/j.biopsych.2015.12.010

Opel, N., Redlich, R., Dohm, K., Zaremba, D., Goltermann, J., Repple, J., … Dannlowski, U. (2019). Mediation of the influence of childhood maltreatment on depression relapse by cortical structure: a 2-year longitudinal observational study. *The Lancet Psychiatry*, *6*(4), 318–326. https://doi.org/10.1016/S2215-0366(19)30044-6

Schwarzmeier, H., Leehr, E. J., Böhnlein, J., Seeger, F. R., Roesmann, K., Gathmann, B., … Dannlowski, U. (2020). Theranostic markers for personalized therapy of spider phobia: Methods of a bicentric external cross-validation machine learning approach. *International Journal of Methods in Psychiatric Research*, *29*(2), e1812. https://doi.org/10.1002/mpr.1812

Vogelbacher, C., Möbius, T. W. D., Sommer, J., Schuster, V., Dannlowski, U., Kircher, T., … Bopp, M. H. A. (2018). The Marburg-Münster Affective Disorders Cohort Study (MACS): A quality assurance protocol for MR neuroimaging data. *NeuroImage*, *172*, 450–460. https://doi.org/10.1016/J.NEUROIMAGE.2018.01.079
